# Determination of insulin resistance and its relationship with hyperandrogenemia,anti-Müllerian hormone, inhibin A, inhibin B, and insulin-like peptide-3 levels in adolescent girls with polycystic ovary syndrome

**DOI:** 10.3906/sag-1808-52

**Published:** 2019-08-08

**Authors:** Aylin YETİM ŞAHİN, Firdevs BAŞ, Çağcıl YETİM, Ahmet UÇAR, Şükran POYRAZOĞLU, Rüveyde BUNDAK, Feyza DARENDELİLER

**Affiliations:** 1 Division of Adolescent Medicine, Department of Pediatrics, İstanbul School of Medicine, İstanbul University, İstanbul Turkey; 2 Division of Pediatric Endocrinology and Diabetes, Department of Pediatrics,İstanbul School of Medicine, İstanbul University, İstanbul Turkey; 3 Department of Gynecology and Obstetrics, School of Medicine, Biruni University, İstanbul Turkey; 4 Division of Pediatric Endocrinology and Diabetes, Department of Pediatrics, Şişli Etfal Training and Research Hospital, İstanbul Turkey

**Keywords:** Adolescent, anti-Müllerian hormone, free androgen index, inhibin A, inhibin B, insulin resistance, polycystic ovary syndrome

## Abstract

**Background/aim:**

This study aims to investigate the association between polycystic ovary syndrome (PCOS) and obesity and insulin resistance (IR) with respect to anti-Müllerian hormone (AMH), inhibin A (INH-A), inhibin B (INH-B), and insulin-like peptide 3 (INSL3) levels, all factors which may have an impact on IR.

**Materials and methods:**

In this cross sectional study, 52 adolescent girls diagnosed with PCOS****[groups: nonobese (NO), n = 23; overweight/obese (OW/O), n = 29] were included. Blood samples were obtained to measure AMH, INH-B, INH-A, and INSL3 levels, together with hormonal and biochemical assessments. Oral glucose tolerance test (OGTT) was performed and the indexes of IR [homeostasis model assessment: insulin resistance (HOMA-IR) and Matsuda index] were calculated.

**Results:**

Insulin resistance was 56.5% with OGTT and 30.4% with HOMA-IR in nonobese-PCOS girls. There was a correlation between INH-A and HOMA-IR even when controlled for body mass index (BMI). INH-B and FAI also had correlations with HOMA-IR which disappeared when controlled for BMI. In regression analyses, AMH (odds ratio = [0.903, P = 0.015) and FAI (odds ratio = 1.353, P = 0.023) are found to be contributors to IR. Their effect was BMI-independent. In ROC analysis, the cutoff value for FAI was 5.93 (sensitivity 71%) to define IR in PCOS girls.

**Conclusion:**

AMH and FAI may contribute to IR (defined by OGTT) in PCOS. FAI might be used as a supporting IR marker (defined by OGTT) in adolescent girls with PCOS.

## 1. Introduction

The frequency of obesity and insulin resistance (IR) is gradually increasing and has a significant association with reproductive endocrinology and menstrual dysregulation, especially in adolescents [1,2]. Obesity and/or IR may lead to an increase in the incidence of polycystic ovary syndrome (PCOS) in adolescents, but may also cause hirsutism and menstrual irregularities even in the absence of PCOS [3,4].

Studies related to new hormones, cytokines, and proteins that help to predict a diagnosis of PCOS and clarify the etiopathogenesis are ongoing [5–10]. A recent study [10] reported that, in addition to anti-Müllerian hormone (AMH), inhibin A (INH-A) could be used as a marker for PCOS. The relationship between these biomarkers and glucose/insulin metabolism in patients with PCOS has been investigated in some studies [8,9,11]. It is known that AMH levels are positively correlated with the number of preantral and small antral follicles [12], and high insulin levels have been found to be associated with antral follicle count and ovarian volume [13]. Recently, a study involving only adolescents and young adults found that the AMH level was elevated in adolescent girls with PCOS who were nonobese and had IR [14]. The relationship between AMH and obesity/IR has been investigated in some studies, but most of them have been conducted with adults and/or have evaluated IR using the homeostasis model assessment: insulin resistance (HOMA-IR) index [11,14–17]. Only a small number of studies have examined glucose/insulin metabolism by way of the clamp test and/or oral glucose tolerance test (OGTT) [17–19]. 

It has been reported that hyperinsulinemia stimulates steroid hormone synthesis in the ovaries, increases the production of androgens, and inhibits the synthesis of steroid hormone binding globulin (SHBG) and IGFBP-1, which binds IGF-1 in the liver [20,21]. Inhibin B (INH-B) and INH-A, released from granulosa cells in the ovaries and involved in follicle development, are also possibly related to IR and/or obesity. It has been suggested that INH-B levels are inversely correlated with body mass index (BMI) in patients with PCOS and that INH-B levels are suppressed by insulin [22,23]. There is limited data about the relation between INH-A levels and insulin secretion [9], and there is no research data from adolescent age groups on this issue. Investigations into the relationship between these biomarkers and IR could illuminate the pathophysiology of PCOS.

A new protein being studied as a possible diagnostic biomarker in patients with PCOS is insulin-like peptide-3 (INSL3), which is released from theca cells of the follicles, the corpus luteum, and ovarian stroma and the levels of which increase at the onset of puberty, like insulin, IGF-1, and IGF-BP3. It has been found to be related to luteinizing hormone (LH)-dependent hyperandrogenism, especially in patients with PCOS who have normal body weights [7]. However, INSL3 was not investigated in adolescent girls with PCOS in terms of IR and/or obesity.

In this study, we aimed to investigate the relationship of obesity/IR with hyperandrogenemia and the levels of specific biomarkers (AMH, INSL3, INH-B, and INH-A) that may have an impact on IR and contribute to emergence of disease in adolescent girls with PCOS. We also aimed to determine the usage areas of these biomarkers in PCOS and contribute to the clarification of its pathophysiology.

## 2. Materials and methods

### 2.1. Study design and subjects

Fifty-two adolescent girls aged 14.5 to 20 years who were consecutively admitted to a pediatric endocrine outpatient clinic between August 2014 and August 2015 were recruited for this cross sectional study. All of the girls had been diagnosed with PCOS based on the Rotterdam criteria. The exclusion criteria are described elsewhere [10]. Written consent was obtained from all participants and their parents. Ethical approval was obtained from the local ethics committee (no. 2421).

### 2.2. Methods

#### 2.2.1. Clinical evaluation

Anthropometric measurements (height, weight, and waist and hip circumference) and scoring of body hair growth using the Ferriman–Gallwey scale [24] were taken for all participants by an experienced physician as described elsewhere [10]. Standard deviation scores (SDS) were calculated according to national data [25–27]. BMI was calculated using the formula BMI = weight (kg)/height (m2). The study group was divided into 2 groups based on BMI: a) the nonobese group (PCOS-NO), who had normal weights (BMI SDS < 1), and b) the overweight (OW) and obese group (PCOS-OW/O), who had been classified as overweight (1–2 BMI SDS) or obese (BMI SDS > 2). Blood pressures (BP) were measured by the same physician, and the SDSs of these measurements were calculated using data from the National High Blood Pressure Education Program Working Group [28].

#### 2.2.2. Laboratory evaluation and biochemical assays

Blood samples for laboratory analysis of glucose, insulin, FSH, LH, SHBG, dehydroepiandrosterone-sulfate (DHEAS), total testosterone (T), androstenedione (D4-A), AMH, INSL3, INH-B, and INH-A were drawn between 8:00 AM and 8:30 AM after an overnight fast. Basal 17-hydroxyprogesterone (17-OHP), free thyroxine, thyroid-stimulating hormone (TSH), cortisol, and prolactin levels were measured to exclude adrenal enzyme defects, thyroid hormone defects, and intracranial pathologies. The blood samples were immediately centrifuged, and the serum samples were stored at −80 °C until assaying. Free androgen index (FAI) was calculated using T and SHBG values in the equation FAI = 100 ×(T/SHBG).

The HOMA-IR index was calculated as (insulin **[**µU/mL] × glucose [mg/dL]) / 405 [29]. HOMA-IR > 2.5 was used to define IR [29]. A standard 75 g OGTT was performed after an overnight fast of at least 8 h but not exceeding 14 h. Venous blood was sampled at 0, 30, 60, 90, and 120 min after glucose intake, and all the venous plasma samples were centrifuged and analyzed for glucose and insulin immediately after blood collection. The whole body insulin sensitivity index (ISIcomp, Matsuda index) was calculated as 10000 / (√ (fasting glucose x fasting insulin) × (mean glucose x mean insulin on OGTT) [30]. Glucose abnormalities were assessed according to American Diabetes Association criteria [31]. Fasting levels of insulin greater than 15 µU/mL, or insulin peak (post-OGTT) levels of more than 150 µU/mL and/or more than 75 µU/mL at 120 minutes of OGTT are hyperinsulinemic levels, which infer IR in adults [32]. The study group was divided into 2 subgroups according to sum of insulin levels during OGTT as follows: a) IR ≥ 300 µU/mL and b) without IR < 300 µU/mL [33].

#### 2.2.3. Assays

Plasma glucose was measured using the hexokinase method with standard equipment and methods (Roche Diagnostics with Cobas Integra kits, Basel, Switzerland). Insulin (IU/mL) was measured using the immunoradiometric assay method (IRMA) (DIA source ImmunoAssays, S.A., Nivelles, Belgium), and the lowest value of detection was 1 IU/mL. Intra- and interassay coefficients of the variation (CVs) were 1.5%–2.1% and 6.1%–6.5%, respectively. Leptin was measured using ELISA with intra- and interassay CVs of < 10% and < 6%, respectively (Abcam Inc, Cambridge, MA, USA). The levels of the other plasma hormones (LH, FSH, SHBG, DHEAS, T, D4-A, AMH, INH-A, INH-B, INSL3, 17-OHP, cortisol, FT4, and TSH) were measured using assays described elsewhere [10].

### 2.3. Statistical analysis

SPSS 15 (IBM, Chicago, IL, USA) was used for statistical analysis. Mean ± SD or median (minimum–maximum) were given for the results. Three different methods (variability coefficient, skewness-kurtosis, and the Kolmogorov–Smirnov values) were used for normality tests of continuous variables, and findings in two of these three methods were considered to confirm normal distribution.

Intergroup comparisons were analyzed using parametric and nonparametric tests. For comparison of categorical variables, the chi-square test was used, and for comparison of continuous variables, Student’s t-test was used. For irregularly distributed parameters, we applied the Mann–Whitney U test, and the Spearman and Pearson correlation parameters were used for correlation analyses. A logistic regression model was applied using a backward step-wise method with the dependent variable of the presence of IR (defined by OGTT) and independent variables of FAI, AMH, INH-A, INH-B, INSL3, leptin, and BMI SDS.

## 3. Results

### 3.1. Clinical findings

There was a higher frequency of acanthosis nigricans and hirsutism in the PCOS-OW/O group than in the PCOS-NO group (P < 0.001 and P = 0.024, respectively). Additionally, as expected, weight SDS, BMI SDS, waist circumference (WC) SDS, and waist/hip ratio (WHR) were higher in the PCOS-OW/O group than in the PCOS-NO group. The systolic BP SDS and diastolic BP SDS values were similar in both groups (data not shown). A comparison of the clinical findings is shown in Table 1.

**Table 1:** Some clinical findings of the patients with PCOS.

	Patients with PCOS(nonobese) n = 23	Patients with PCOS(obese) n = 29	P-value
Age (years)	16.84 ± 1.36	16.64 ± 1.48	0.608
Menarcheal age (yrs)	12.59 ± 1.50	12.50 ± 1.27	0.822
BW SDS	−0.38 [−2.30 to 3.79]	0.19 [−3.66 to 3.11]	0.793
Height SDS	−0.48 [−3.30 to 1.58]	0.00 [−2.33 to 2.89]	0.056
Weight SDS	−0.56 [−2.88 to 1.11]	2.33 [0.91 to 5.41]	<0.001
BMI SDS	−0.60 [−1.74 to 0.98]	2.39 [1.28 to 9.20]	<0.001
WC SDS	1.01 [0.06 to 3.63]	4.98 [2.93 to 10.47]	<0.001
WHR	0.83 ± 0.05	0.89 ± 0.05	<0.001

PCOS: Polycyctic ovary syndrome, SDS: Standard deviation score, BW: Birth weight, SGA: Small for gestational age, AGA: Appropriate for gestational age, LGA: Large for gestational age, BMI: Body Mass Index, WC: Waist circumference, WHR: Waist-hip ratio

### 3.2. Glucose and lipid metabolism

The HOMA-IR index level was higher and the Matsuda index value was lower in the PCOS-OW/O group than in the PCOS-NO group (P = 0.001 and P = 0.002, respectively). The rate of IR detected by HOMA-IR in the PCOS-NO group was significantly lower than the rate detected by OGTT (30.4% and 56.5%, respectively), but there were no significant differences in the PCOS-OW/O group (72.4% and 79.3%, respectively). The detection of glucose intolerance was similar in the PCOS-NO and PCOS-OW/O groups (8.7% and 6.9%, respectively) (Table 2), and no patient fulfilled the criteria for metabolic syndrome. In terms of lipid metabolism, all parameters were similar between the two groups (data not shown). 

**Table 2:** Laboratory findings of the patients with PCOS regarding glucose metabolism.

	Patients with PCOS(nonobese) n = 23	Patients with PCOS(obese) n = 29	P-value
Fasting glucose (mg/dL)	69.87 ± 6.38	71.45 ± 8.94	0.478
Fasting insulin (µU/mL)	11.64 ± 6.23	24.48 ± 16.83	0.001
2-h glucose (mg/dL)	108.86 ±27.47	102.93 ± 27.61	0.314
Glucose intolerance (2-h glucose) n(%)	2 (8.7)	2 (6.9)	0.806
Mean serum insulin (µU/mL)	86.75 ± 64.79	110.05 ± 60.02	0.196
Matsuda index	4.98 ± 2.43	3.06 ± 1.74	0.002
HOMA-IR	2.04 ± 1.16	4.35 ± 3.00	0.001
Presence of IR (with HOMA-IR) n(%)	7 (30.4)	21 (72.4)	0.004
Presence of IR (with OGTT) n(%)	13 (56.5)	23 (79.3)	0.071

PCOS: Polycyctic ovary syndrome, IR: Insulin resistance, HOMA-IR: Homeostasis model assessment-insulin resistance, OGTT: Oral glucose tolarence test, SDS: Standard deviation score.

### 3.3. Hormone profile

SHBG level was statistically significantly lower, and the D4-A and FAI were higher, in the PCOS-OW/O group than in the PCOS-NO group. There were no differences shown in INH-A between the PCOS-OW/O group and the. PCOS-NO group. INSL3 and INH-B levels were statistically significantly lower in the PCOS-OW/O group than in the PCOS-NO group. A comparison of hormone levels between the groups is presented in Table 3. 

**Table 3:** Some laboratory findings of the patients with PCOS.

	Patients with PCOS(nonobese) n = 23	Patients with PCOS(obese) n = 29	P-value
LH (mU/mL)	10.60 [4.70–79.10]	10.60 [3.00–25.80]	0.755
FSH (mU/mL)	5.00 ± 1.83	5.41 ± 1.69	0.419
LH/FSH ratio	3.23 ± 2.58	2.19 ± 0.89	0.077
D4-A (ng/mL)	4.03 ± 1.43	5.75 ± 2.32	0.004
DHEA-S (µg/dL)	284.60 ± 118.14	314.13 ±127.75	0.396
T (ng/mL)	0.44 [0.19– 1.03]	0.47 [0.16–2.21]	0.536
SHBG (ng/mL)	13.23 ± 8.64	7.19 ±4.81	0.008
FAI [100 × (T/SHBG)]	3.35 [0.97–13.04]	8.67 [2.30–23.65]	0.002
Leptin (ng/mL)	6.24 ± 2.98	11.23 ± 5.81	<0.001
AMH (ng/mL)	12.36 ± 9.06	18.07 ± 13.14	0.071
INH-A (pg/mL)	28.50 [1.76–239.70]	30.54 [1.68–307.10]	0.692
INH-B (pg/mL)	92.28 ± 63.44	54.74 ± 30.97	0.014
INSL3 (pg/mL)	1353.69 ± 289.19	1179.69 ± 264.58	0.028

Data is expressed as either number, mean ± SD or median (min-max) for variables showed abnormal distribution. PCOS: Polycyctic ovary syndrome, LH: Luteinizing hormone, FSH: Follicle-stimulating hormone, T: Testosterone, SHBG: Sex hormone binding globulin, FAI: Free androgen index, DHEAS: dehydroepiandrosterone-sulphate, DA-4: Androstenedione, AMH: Anti-Müllerian hormone, INH-A: Inhibin A, INH-B: Inhibin B, INSL3: Insulin-like peptide-3.

### 3.4. Correlation analysis

Luteinizing hormone, FSH, total T, DHEAS, AMH, and INH-A showed no correlations with anthropometric measurements. BMI SDS was significantly correlated with FAI, D4-A, leptin, SHBG, and INH-B (r = 0.519, P < 0.001; r = 0.372, P = 0.011; r = 0.612, P < 0.001; r = −0.525, P < 0.001; and r = −0.368, P = 0.007, respectively), and WC SDS showed similar correlations.

Inhibin B was significantly correlated with both the HOMA-IR index and the Matsuda index without controlling for BMI SDS. However, in partial correlation analysis performed by removing the BMI effect, INH-B was only correlated with the Matsuda index (r = 0.291, P = 0.044). Inhibin A was significantly correlated with HOMA-IR even after controlling for the BMI effect (r = −0.324, P = 0.025). Anti-Müllerian hormone had no correlation with HOMA-IR or the Matsuda index with or without BMI effect. SHBG had a negative correlation with HOMA-IR, but no correlation was found after controlling for BMI. And no correlation was found between SHBG and the Matsuda index. FAI was significantly correlated with both HOMA-IR and the Matsuda index, but in partial correlation analysis performed by removing BMI effect, these correlations disappeared. The correlations of hormone levels, and FAI with HOMA-IR and the Matsuda index (with and without BMI effect) are shown in Table 4.

**Table 4:** Correlations of hormone levels and FAI with HOMA-IR and Matsuda index (with and without BMI effect) in the patients with PCOS.

With BMI effect (not controlled for BMI)	HOMA-IR		Matsuda index	
	r	P	r	P
D4-A (ng/mL)	0.416	0.004	−0.246	0.111
T (ng/mL)*	0.068	0.635	−0.241	0.099
SHBG (ng/mL)	−0.323	0.027	0.296	0.051
FAI*	0.358	0.014	−0.531	<0.001
Leptin (ng/mL)	0.496	<0.001	−0.439	0.002
AMH (ng/mL)	−0.066	0.641	0.182	0.212
INH-A (pg/mL)*	−0.284	0.042	0.272	0.059
INH-B (pg/mL)	−0.386	0.005	0.429	0.002
INSL3 (pg/mL)	0.187	0.186	−0.103	0.480
Without BMI effect (controlled for BMI)	HOMA-IR		Matsuda index	
	r	P	r	P
D4-A (ng/mL)	0.217	0.167	-0.073	0.648
T (ng/mL)*	0.031	0.838	−0.126	0.397
SHBG (ng/mL)	0.013	0.934	0.043	0.783
FAI*	0.253	0.106	−0.246	0.117
Leptin (ng/mL)	−0.038	0.797	−0.159	0.282
AMH (ng/mL)	−0.119	0.421	0.220	0.133
INH-A (pg/mL)*	−0.324	0.025	0.247	0.091
INH-B (pg/mL)	−0.147	0.318	0.291	0.044
INSL3 (pg/mL)	0.301	0.038	−0.134	0.365

*Spearman correlation analysis was used for these irregularly scattered parameters. PCOS: Polycyctic ovary syndrome, HOMA-IR: Homeostasis model assessment-insulin resistance, SDS: Standard deviation score, T: Testosterone, SHBG: Sex hormone binding globulin, FAI: Free androgen index, DA-4: Androstenedione, AMH: Anti-Müllerian hormone, INH-A: Inhibin A, INH-B: Inhibin B, INSL3: Insulin-like peptide-3.

### 3.5. Regression analysis

In logistic regression analysis performed with the dependent variable of IR presence (defined using OGTT) and the independent variables of FAI, AMH, INH-A, INH-B, INSL3, leptin, and BMI SDS; the variables of INH-A, INH-B, INSL3, and also BMI SDS were excluded through the steps. The significant contributors of IR were FAI [odds ratio (95% Cl) = 1.353 (1.042–1.756), P = 0.023], and AMH [odds ratio (95% Cl) = 0.903 (0.832–0.981), P = 0.015].

### 3.6. Receiving operating curve (ROC) analysis

FAI was found to be an IR marker (defined using OGTT) at the cutoff value of 5.93 with 71% sensitivity and 67% specificity (area under curve: 69.9%; P = 0.03; 95% CI= 0.546–0.852) (Figure). In ROC analysis performed for AMH to be an IR marker, sensitivity and specificity were low (at the cutoff value of 10, 50% sensitivity, 69% specificity, area under curve: 32.6%; P = 0.047; 95% CI = 0.171–0.482). 

**Figure:**
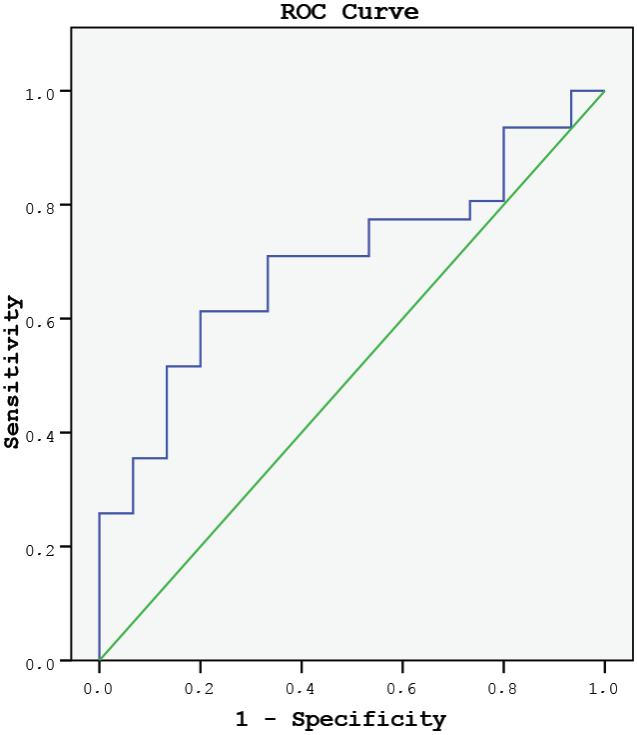
ROC analysis for FAI to define the cutoff values that can be used as an insulin resistance sign.

## 4. Discussion

The role of specific biomarkers (AMH, INH-A, INH-B, INSL3) and hyperandrogenemia in pathophysiology of PCOS and the association of them with obesity and/or IR have been investigated in different studies; however, to the best of our knowledge, this study is the only one that analyzed the association of all these biomarkers and hyperandrogenemia together with obesity and IR, determined IR by OGTT, and restricted the participants to only adolescent girls. We found AMH and FAI but not BMI as contributors to IR defined by OGTT, and we offered a cutoff value for FAI to be used as an IR marker in PCOS. 

In our study, the frequency of IR was found to be high in both overweight/obese and nonobese girls with PCOS. In the PCOS-NO group, the rate of IR detection by OGTT was significantly higher than by HOMA-IR. Similarly, Flannery et al. [34] proposed that glucose/insulin metabolism should be evaluated in detail in patients with PCOS independently of BMI, and that nonobese patients with PCOS should be investigated in terms of impaired glucose tolerance and IR. Coles et al. [35] reported that OGTT was a superior diagnostic tool in assessing glucose metabolism disorder. In light of our study’s results and current publications, it is clear that patients with PCOS should be evaluated for IR in detail. 

Our study showed a similar frequency of impaired glucose tolerance in the PCOS-OW/O and PCOS-NO groups. There were no patients with PCOS who met the criteria for metabolic syndrome; however, a study conducted by Hart et al. [36] reported that the risk of developing metabolic syndrome was high in adolescents with PCOS. Therefore, physicians should be alert in this respect during patient follow-up visits.

In a metaanalysis that investigated the relationship between IR and androgen levels in patients with PCOS, it was found that SHBG had a strong association with IR that was not confounded by BMI, and total T had a moderate effect on IR [19]. Flannery et al. [34] showed that nonobese patients with PCOS with impaired glucose tolerance had similar T levels to those of obese patients. In a study conducted by Reyes-Munoz et al. [37], which sampled a large number of patients, it was reported that total T levels were not different between overweight/obese patients and normal weight patients with PCOS, whereas significant differences in SHBG and FAI were observed. We found a negative association between SHBG and IR in our study, but when controlled for BMI, this association disappeared. There was no relationship between total T levels and obesity/IR parameters. However, consistent with the literature, FAI was found to be more valuable than using T or SHBG alone for monitoring IR in PCOS, as it stayed as an effective factor on IR together with AMH in regression analysis. Also FAI might be used as a supporting IR marker at the cutoff value of 5.93, according to ROC analysis**.**

INH-A was not found to be related to IR in a study conducted by Segal et al. [9]; however, IR was evaluated only by fasting insulin level in this study. For INH-B, some studies have shown a negative association between INH-B and insulin levels [22,23]. We found a negative correlation between INH-A and HOMA-IR, even when controlled for BMI. INH-B also had a negative correlation with HOMA-IR, but the correlation disappeared when controlled for BMI. However neither INH-A nor INH-B contribute to IR (defined by OGTT) in regression analysis. According to our results, INH-B is related to BMI, but more research is needed to clarify the relationship of INH-A and INH-B with IR in PCOS.

Studies conducted with PCOS patients emphasize that there is an inverse correlation between BMI and AMH [38–41]. However, these studies have been conducted with adults. In a study conducted with adolescent girls with PCOS, no difference was reported in AMH levels between the groups with normal weight and the overweight/obese patients [18]. Similarly, we found no relation between AMH levels and BMI. 

Examination of the association between obesity/IR parameters and AMH levels will provide a clearer interpretation of the pathophysiology of PCOS. It has been shown that AMH triggers hyperandrogenism and IR in patients with PCOS [42]. There are some contradictory results on the relationship between AMH and IR in patients with PCOS. Skalba et al. [40] found a positive correlation, while Feldman et al. [41] found a negative correlation between AMH and IR. However, in the study conducted by Skalba et al. [40], PCOS group consisted only of patients with normal and overweight BMIs, whereas Feldman et al. [41] also included obese patients with PCOS. In two studies with a high number of PCOS patients conducted by Cui et al. [38, 39] , AMH was found to be not related to glucose/insulin metabolism and IR. However, in these studies, the relationship between IR and AMH were examined by HOMA-IR. In our study, IR was evaluated by both HOMA-IR and OGTT. AMH did not have a significant effect on IR in a regression model when studied alone, but did when combined with FAI when BMI was excluded from the equation. We urge that the relationship between AMH and IR be investigated with studies on a larger number of patients using OGTT or the clamp test. 

The present study presenting the preliminary results of our ongoing work was conducted with a relatively limited number of patients. Another limitation was the absence of a control group due to the fact that no ethical approval could be obtained for performing OGTT in a control group. 

In conclusion, OGTT is more reliable than HOMA-IR in specifying IR in nonobese adolescent girls with PCOS. Inhibin B is associated with BMI rather than IR. INH-A is associated with HOMA-IR and this association is BMI-independent. Similarly, AMH and FAI together may contribute to IR defined by OGTT and this contribution is also BMI-independent. FAI can be used as a supporting IR marker in PCOS. 

## Acknowledgments

This work was supported by Scientific Research Projects Coordination Unit of İstanbul University (Project no. 47791). The authors would like to thank Mrs. Eve M. Richards for editing the manuscript.
